# Stenting deferral in primary percutaneous coronary intervention: exploring benefits and suitable interval in heavy thrombus burden

**DOI:** 10.1186/s43044-021-00203-3

**Published:** 2021-09-09

**Authors:** Ahmed M. Magdy, Salwa R. Demitry, Hosam Hasan-Ali, Mohamed Zaky, Mohamed Abd El-Hady, Mohamed Abdel Ghany

**Affiliations:** 1 Cardiovascular Medicine, National Heart Institute, Cairo, Egypt; 2 Cardiovascular Medicine, Faculty of Medicine, Assiut University, Asyut, Egypt

**Keywords:** Deferral, ST-segment elevation, Myocardial infarction, Slow-flow, No-reflow

## Abstract

**Background:**

Deferred stenting, despite being successful in early studies, showed no benefit in recent trials. However, these trials were testing routine deferral; not in patients with heavy thrombus burden.

**Results:**

This is a prospective, Randomized Clinical Trial that included 150 patients who presented with STEMI, patients were allocated into three equal groups after the coronary angiography ± primary intervention and before stenting of the culprit lesion; group (A) included 50 patients with early deferral of stenting, group (B) included 50 patients with late deferral and group (C) included 50 patients with immediate stenting. No-reflow was significantly higher in group C, while Final TIMI flow grade 3 and MBG grade 3 were significantly higher in group A and B than group C; *p* = 0.019 and < 0.001 respectively, with no significant difference between groups A and B, only the thrombus resolution in group B was significantly higher than group A; *p* < 0.001. Finally, 6-months, over-all MACE was significantly higher in group C (34.7% vs. 14.6% and 16.3%, *p* = 0.029).

**Conclusions:**

Stent deferral was proved to be better than immediate stenting after recanalization of IRA, in achieving TIMI III flow, reducing risk of 6 months MACE, and restoration of myocardial function in a subset of STEMI patients presenting with large thrombus burden. While, no significant difference was found between both deferral times in final TIMI flow, or clinical outcomes.

## Background

Primary percutaneous coronary intervention (PCI) with stent implantation is the current standard treatment for patients with ST-segment elevation myocardial infarction (STEMI) [1]. However, even when revascularization of the occluded vessel appears successful, distal embolization can occur, which leads to microvascular dysfunction, and is associated with an unfavorable prognosis [2]. Therefore, a considerable number of patients with STEMI treated with primary PCI shows reduction in blood flow in the culprit artery, that usually occurs after stent implantation. Even in patients with normal epicardial flow, myocardial perfusion may be impaired [3, 4]

Distal embolization during lesion manipulation was thought to be a major cause of no-reflow, yet, using distal protection devices didn’t prove to be of value [5, 6], and thrombectomy seems beneficial in some settings, but in others it may increase infarct size [7, 8].

Stent implantation timing remains a controversial issue, as immediate stenting in a thrombotic context is associated with many risks [9]. Immediate stenting was found to be associated with no-reflow, distal embolization by thrombus, and impairment of myocardial perfusion [10–12]. This has led investigators to defer stenting when artery patency was obtained. This strategy was proposed in some earlier studies with some success [13, 14]. The deferred stenting after restoration of normal epicardial flow by immediate intervention (MIMI) by Isaac et al. [13] proposed the idea of stenting deferral in STEMI. The idea that was explored by other studies afterwards, with better procedural end results, short and long-term results as better left ventricular function and lower incidence of MACE in comparison with immediate stenting [13, 15–17]. Carrick et al. [16], found that deferred stenting in primary PCI reduced no-reflow and increased myocardial salvage compared with conventional primary PCI with immediate stenting. Randomization was to either perform immediate stenting or deferral of stenting after recanalization by 4–16 h interval [16]. Also, the INNOVATION trial showed that patients randomly assigned to stenting 3 to 7 days after their initial procedure for STEMI “had a strong tendency” toward reduction in infarct size, as well as reduced microvascular obstruction (MVO) versus those managed with immediate stenting [18].

In contrast, recent results from new randomized controlled trials (RCTs), showed some inconsistent results compared with previous observational studies. However, these trials were testing routine deferral and not in a special subset [19, 20]. To get a clearer view of this controversial issue, our study aimed to investigate the beneficial deferral time of stenting in 1ry PCI after recanalization of the infarct related artery in a specific subset of patients presenting with heavy thrombus burden who are expected to have a higher risk of no-reflow with immediate stenting. Aiming to gain benefit from the fact that delayed stenting may carry the following advantages; decreasing rates of angiographic events (distal emboli, no-reflow) with reduced infarct size which gives time to assign the most appropriate treatment strategy (stent vs. coronary artery bypass grafting vs. medical therapy alone), noting that neither stent nor PTCA were needed in 10% of patients allowing statin preloading before angioplasty in addition to the possible reduction in congestive heart failure, re-infarction and death [21–23].

## Methods

This is a prospective, Randomized Clinical Trial that included 150 patients who presented with STEMI. The study was held in the period from 2016 to 2019.

Block Randomization was used in this study; prior to the beginning of the study, a simple random sample through Microsoft Excel 2010 was done to randomly select one of the sequences of block of 3 (A, B, C) to determine intervention that will be received by the first three patients eligible for the study, then the following sequences of block were randomly selected the same way and this was repeated till 50 blocks of 3 were prepared for the 150 patients eligible for the study. Patients were allocated accordingly into three equal groups after the coronary angiography ± primary intervention (in order to achieve TIMI II-III flow in IRA either spontaneously, by aspiration thrombectomy or by balloon dilatation) and before stenting of the culprit lesion; Group (A) included 50 patients with intention to stent the residual stenosis 4–16 h later (Early deferral). Group (B) included 50 patients with intention to stent the residual stenosis after 7 days (Late deferral). Group (C) included 50 patients treated with the standard clinical practice of immediate stenting. Blinding was non-applicable with the implemented intervention.

The included patients fulfilled the following; STEMI with symptoms of ischemia of ≤ 12 h duration, OR time from symptom onset > 12 h in the presence of ongoing symptoms suggestive of ischemia, or life-threatening arrhythmias, had a heavy thrombus burden in the infarct related artery (2–5 TIMI thrombus grade), and achievement of TIMI II-III flow in IRA before randomization. Patients were excluded if presented with cardiogenic shock, culprit lesions in unprotected left main coronary artery or saphenous vein grafts, failure to achieve TIMI flow grade II-III before randomization to either immediate stenting or deferral, patients with type C dissections or worse in IRA, patients who had previous myocardial infarction in the target vessel area, patients with stent thrombosis, and evidence of GIT bleeding within 1 month.

All patients were subjected to full history taking and data recording including; age, sex, risk factors like diabetes, hypertension, smoking (either former or current), positive family history for ischemic heart disease, and/or prior angina, PCI and CABG. Thorough physical examination including vital signs (pulse and blood pressure), complete general examination and local cardiac examination were performed for every patient. Resting ECG was performed paying special attention for detection of electrocardiographic criteria of STEMI, and detecting arrhythmias.

Admission serum glucose level, serum creatinine and cardiac enzymes were measured. In addition to standard routine laboratory investigation.

An echocardiogram was performed on first day of hospitalization and after 6 months, and the differences in results between both studies were compared regarding left ventricular function.

### Interventions

Patients received 300 mg aspirin, and loading dose of ticagrelor prior to coronary angiography, with heparin given before intervention. Coronary angiography was performed in the standard fashion; through femoral approach. Selective coronary angiography was performed. Angiographic data of the patients were obtained, which included an average of six left coronary and two right coronary artery injections giving sufficient data to enable quantitative angiography and identification of the culprit lesion [24].

After coronary angiography and inclusion of the patient due to heavy thrombus criteria, TIMI III flow was pursued in IRA (either spontaneous, using balloon angioplasty or aspiration catheter). Direct stenting was the preferred strategy, with pre-dilatation only performed when necessary. In the deferred stenting group, TIMI II-III was accepted at the end of the 1st procedure and PCI was performed after intensive pharmacologic treatment for 4–16 the specified deferral time.

The treatment protocol for deferred patients included transferring to the Coronary Care Unit, continuous intravenous infusion of glycoprotein IIb/IIIa inhibitor for 48 h (Irrespective of time of deferral of stenting), administration of subcutaneous low molecular weight heparin (enoxaparin 1 mg/kg every 12 h until the 2nd procedure), and double antiplatelet therapy with aspirin and ticagrelor, in addition to evidence based medical treatment according to current guidelines [25].

### Assessment of microvascular perfusion

TIMI flow grade system and myocardial blush grade (MBG) were used for assessment by 2 physicians in a blinded manner [26].

### Follow-up

Clinical data were collected for patients in pre-specified visits to the outpatient clinic and using telephone interviews in-between. During the initial hospital stay, the following were evaluated:

1. Incidence of Major Adverse Cardiovascular Events (MACE) including cardiac death, nonfatal re-infarction (when ST-elevation ≥ 1 mm recurs or new pathognomonic Q waves appear in at least two contiguous leads, particularly when associated with ischemic symptoms, and requires more than 20% increase of the cTn value in the second sample) [27], arrhythmias, heart failure (defined by auscultation of 3rd heart sound, NYHA class III or more, dyspnea, or a chest X-ray denoting pulmonary congestion) and nonfatal cerebrovascular stroke.
2. The occurrence of CIN (an increase of *25%* or more, or an absolute increase of *0.5 mg/dl* or more in serum creatinine from baseline value, at *48–72 h* following the exposure to *contrast media)* [28]*.*
3. Bleeding (requiring transfusion).

While the occurrence of MACE was re-evaluated at 6-months follow-up as well as echocardiographic evaluation.

### Definitions of endpoints

The primary endpoint was the incidence of no-reflow, defined as absence of flow (TIMI flow 0), incomplete filling (TIMI flow I), complete filling with slow flow (TIMI flow II), of the infarct related artery at the end of the procedure as evident angiographically. The secondary endpoint included angiographic parameters (i.e. MBG), echocardiographic parameters (i.e. EF by Simpson’s method) and occurrence of MACE.

### Statistical analysis

Analysis of data was done using Statistical Program for Social Science version 24. Quantitative variables were presented in the form of mean and standard deviation. Continuous variables were compared using Student’s T test for independent groups which was used in case of normal distribution or Mann Whitney test as a non-parametric alternative. Qualitative variables were described as number and percent. Qualitative variables were compared using chi-square (*X*^2^) and Fisher exact test, as indicated. A *p* value less than 0.05 was considered statistically significant.

## Results

This study included 150 patients with mean age 51.5 ± 10.5 years; of whom males represented 77.3%. Regarding the studied risk factors; almost half of the patients were smokers and hypertensive (54% and 49.3% respectively), nearly one third of the patients were diabetic (31.3%), 27.3% had dyslipidemia and 9.3% of the studied sample had history of IHD. More than half of the patients were diagnosed with anterior STEMI (53.3%), 44% Inferior STEMI and 2.7% Lateral STEMI. Median heart rate of the patients was 96 beats/min and their median BP was 120/70 mmHg. As for Killip classification; more than half of the patients were classified in class 1 (54%), 38.7% class 2 and 7.3% class 3 as shown in Table 1.

**Table 1:** Baseline characteristics of the whole study population

Baseline characteristics	All patients (*N* = 150)
Age (years)	51.5 ± 10.5
Risk factors	
Male gender	116 (77.3%)
DM	47 (31.3%)
HTN	74 (49.3%)
Smoking	81 (54%)
Dyslipidemia	41 (27.3%)
IHD	14 (9.3%)
Diagnosis	
Anterior STEMI	80 (53.3%)
Inferior STEMI	66 (44%)
Lateral STEMI	4 (2.7%)
Heart rate (beat/min)	96 (90–110)
SBP (mmHg)	120 (100–130)
DBP (mmHg)	70 (60–80)
Killip class	
Class 1	81 (54%)
Class 2	58 (38.7%)
Class 3	11 (7.3%)

No significant difference was found between the studied groups regarding the baseline characteristics (age, gender, risk factors), diagnosis, clinical data (heart rate, SBP, DBP and Killip classification), baseline echocardiographic data (EF) and creatinine level (*p* > 0.05) as shown in Table 2.

**Table 2:** Comparison between the studied groups regarding the baseline data

Clinical data	Group A (*N* = 50)	Group B (*N* = 50)	Group C (*N* = 50)	*p* value
Age (years)	50.8 ± 9.8	49.8 ± 10.3	54.0 ± 11.0	0.117
Risk factors				
Male gender	37 (74%)	38 (76%)	41 (82%)	0.610
DM	16 (32%)	14 (28%)	17 (34%)	0.805
HTN	27 (54%)	22 (44%)	25 (50%)	0.602
Smoking	28 (56%)	27 (54%)	26 (52%)	0.923
Dyslipidemia	15 (30%)	11 (22%)	15 (30%)	0.584
IHD	3 (6%)	6 (12%)	5 (10%)	0.576
Diagnosis				
Anterior STEMI	19 (38%)	29 (58%)	32 (64%)	0.111
Inferior STEMI	29 (58%)	20 (40%)	17 (34%)	
Lateral STEMI	2 (4%)	1 (2%)	1 (2%)	
Heart rate (beat/min)	95 (90–110)	95 (90–110)	98 (90–110)	0.699
SBP (mmHg)	117.5 (100–130)	120 (100–130)	120 (100–130)	0.904
DBP (mmHg)	70 (60–80)	75 (60–80)	75 (60–80)	0.840
Killip class				
Class 1	29 (58%)	26 (52%)	26 (52%)	0.342
Class 2	19 (38%)	20 (40%)	19 (38%)	
Class 3	2 (4%)	4 (8%)	5 (10%)	
Baseline EF (%)	40 (38–46)	40 (38–47)	40 (38–47)	0.831
Serum Creatinine (mg/dL)	1.0 (0.9–1.1)	1.0 (0.9–1.2)	1.0 (0.9–1.2)	0.779

Comparison between the studied groups regarding angiographic and 1st procedural data demonstrated that there was no significant difference between the 3 groups regarding ‘IRA’, thrombus grade and baseline TIMI flow, (*p* values > 0.05). After the 1st procedure; the percentage of patients with TIMI flow grade 3 was significantly higher in groups A and B compared to group C (90% and 92% Vs. 76%, *p* value = 0.034), however no significant difference was found regarding the procedure itself as well as the myocardial blush grade ‘MBG’ (*p* values > 0.05) as shown in Table 3.

**Table 3:** Comparison between the studied groups regarding the angiographic and procedural data of 1st procedure

Angiographic and procedural data of 1st procedure	Group A (4–16 h deferral) Number = 50	Group B (7 days deferral) Number = 50	Group C (immediate) Number = 50	*p* value
IRA				
LAD	19 (38%)	29 (58%)	32 (64%)	0.059
LCX	9 (18%)	3 (6%)	5 (10%)	
RCA	22 (44%)	18 (36%)	13 (26%)	
Thrombus grade at 1st procedure				
Grade 2	2 (4%)	0 (0%)	3 (6%)	0.407
Grade 3	2 (4%)	4 (8%)	9 (18%)	
Grade 4	35 (70%)	25 (50%)	24 (48%)	
Grade 5	11 (22%)	21 (42%)	14 (28%)	
Baseline TIMI flow				
TIMI 0	35 (70%)	29 (58%)	33 (66%)	1.000
TIMI 1	3 (6%)	12 (24%)	6 (12%)	
TIMI 2	8 (16%)	7 (14%)	8 (16%)	
TIMI 3	4 (8%)	2 (4%)	3 (6%)	
Procedural data				
Balloon angioplasty	30 (60%)	32 (64%)	32 (64%)	0.892
Aspiration	12 (24%)	16 (32%)	11 (22%)	0.483
PCI to non-culprit	11 (22%)	10 (20%)	5 (10%)	0.236
TIMI flow after 1st procedure				
TIMI 0	0 (0%)	0 (0%)	6 (12%)	0.034
TIMI 1	0 (0%)	0 (0%)	3 (6%)	
TIMI 2	5 (10%)	4(8%)	3 (6%)	
TIMI 3	45 (90%)	46 (92%)	38 (76%)	
MBG after 1st procedure				
Grade 0	19 (38%)	14 (28%)	27 (54%)	0.224
Grade 1	12 (24%)	18 (36%)	9 (18%)	
Grade 2	12 (24%)	12 (24%)	7 (14%)	
Grade 3	7 (14%)	6 (12%)	7 (14%)	

Regarding the angiographic data of the 2nd procedure; comparison between groups A and B in-hospital survivors (*n* = 97; group *A* = 48 and group *B* = 49 survivors) revealed that the thrombus resolution in group B (late deferral) was significantly better than group A (early deferral); *p* < 0.001, however there was no significant difference between the two groups regarding the TIMI flow as well as the MBG; *p* > 0.05 as shown in Table 4.

**Table 4:** Comparison between the studied groups regarding the angiographic data of the 2nd procedure of groups A & B in-hospital survivors (*n* = 97)

Angiographic data of 2nd procedure	Group A (4–16 h deferral) Number = 48	Group B (7 days deferral) Number = 49	*p* value
Thrombus grade at 2nd procedure			
Grade 0	3 (6.3%)	14 (28.7%)	< 0.001
Grade 1	1 (2.1%)	6 (12.2%)	
Grade 2	21 (43.7%)	25 (51%)	
Grade 3	14 (29.2%)	1 (2%)	
Grade 4	4 (8.3%)	2 (4.1%)	
Grade 5	5 (10.4%)	1 (2%)	
TIMI flow after 2nd procedure			
TIMI 2	10 (20.8%)	7 (14.3%)	0.396
TIMI 3	38 (79.2%)	42 (85.7%)	
MBG after 2nd procedure			
Grade 0	4 (8.3%)	3 (6.1%)	0.892
Grade 1	7 (14.6%)	7 (14.3%)	
Grade 2	11 (22.9%)	9 (18.4%)	
Grade 3	26 (54.2%)	30 (61.2%)	

Comparison between the studied groups showed that median lesion length was significantly higher in group C compared to both groups A and B (24 vs. 20.5, *p* = 0.037 and 24 vs. 20, *p* = 0.004 respectively), however no significant difference was found between groups A and B; *p* > 0.05, also regarding the angiographic outcome; after the 1st procedure incidence of no-reflow was significantly higher in group C than groups A and B (24% vs. 10% and 8%, *p* = 0.043) while after the 2nd procedure the incidence of no-reflow was matched between groups A and B; *p* > 0.05). Final TIMI flow grade 3 and MBG grade 3 were significantly higher in group A and B than group C; *p* = 0.019 and < 0.001 respectively as shown in Table 5.

**Table 5:** Comparison between the studied groups regarding the final lesion length and angiographic outcome

Lesion length and angiographic outcome		Group A (4–16 h deferral) Number = 50 (48 for 2nd procedure)	Group B (7 days deferral) Number = 50 (49 for 2nd procedure)	Group C (immediate) Number = 50	*p* value
Lesion length (mm)		20.5 (18–24)	20 (16–25)	24 (20–27)	0.003
No-reflow after 1st procedure		5 (10%)	4 (8%)	12 (24%)	0.043
No-reflow after 2nd procedure		10 (20.8%)	7 (14.3%)	–	0.396
Final TIMI flow					
TIMI 0		0 (0%)	0 (0%)	6 (12%)	0.019
TIMI 1		0 (0%)	0 (0%)	3 (6%)	
TIMI 2		10 (20.8%)	7 (14.3%)	3 (6%)	
TIMI 3		38 (79.2%)	42 (85.7%)	38 (76%)	
Final MBG					
Grade 0		4 (8.3%)	3 (6.1%)	27 (54%)	< 0.001
Grade 1		7 (14.6%)	7 (14.3%)	9 (18%)	
Grade 2		11 (22.9%)	9 (18.4%)	7 (14%)	
Grade 3		26 (54.2%)	30 (61.2%)	7 (14%)	

**Fig. 1:**
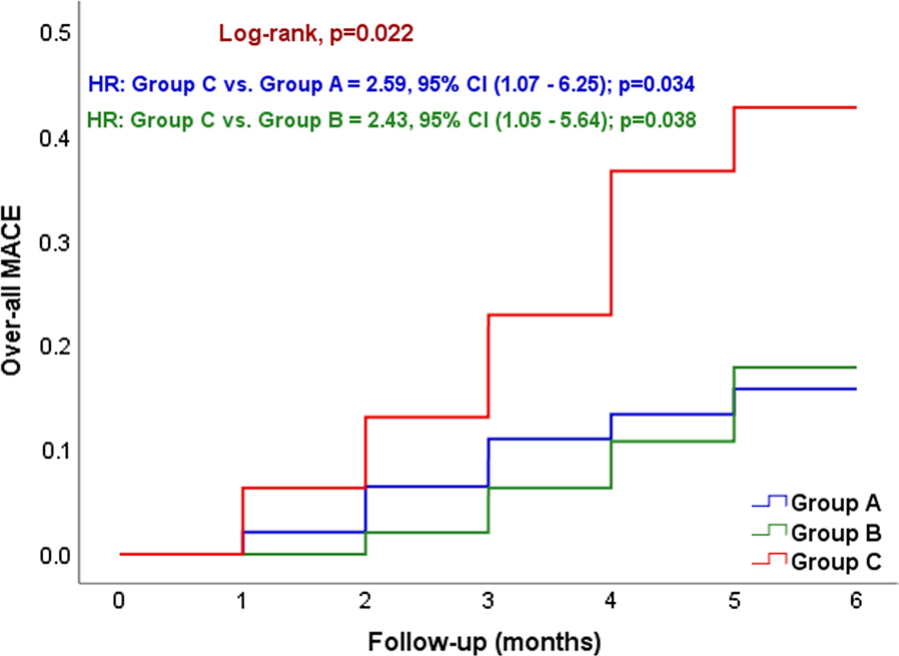
Kaplan Mier survival analysis with hazard proportionate function showing the incidence rate of over-all MACE among groups A, B and C. *MACE* major adverse cardiovascular events

Comparison between the studied groups regarding the in-hospital outcome showed that there was no significant difference between the 3 groups regarding over-all MACE, bleeding and CIN; *p* > 0.05. While, after 6-months, over-all MACE was significantly higher in group C than groups A and B (34.7% vs. 14.6% and 16.3%, *p* = 0.029), the most frequent event reported in each of groups A and C was heart failure (8.3% and 14.3%) and in group B was Non-fatal MI (8.2%) as shown in Table 6 and illustrated by Kaplan Mier curve in Fig. 1.

**Table 6:** Comparison between the studied groups regarding the clinical outcome

Clinical outcome	Group A (4–16 h deferral)	Group B (7 days deferral)	Group C (immediate)	*p* value
In-hospital outcome	Number = 50	Number = 50	Number = 50	
Over-all MACE	9 (18%)	8 (16%)	10 (20%)	0.873
Death	2 (4%)	1 (2%)	1 (2%)	0.773
Re-infarction	3 (6%)	3 (6%)	6 (12%)	0.443
Arrhythmias	5 (10%)	5 (10%)	3 (6%)	0.714
Cardiac decompensation	1 (2%)	2 (4%)	2 (4%)	0.813
Stroke or TIA	1 (2%)	1 (2%)	1 (2%)	1.000
Bleeding	3 (6%)	3 (6%)	2 (4%)	0.876
CIN	4 (8%)	5 (10%)	4 (8%)	0.919

6-month outcome	Number = 48	Number = 49	Number = 49	
Over-all MACE	7 (14.6%)	8 (16.3%)	17 (34.7%)	0.029
Death	1 (2.1%)	2 (4.1%)	3 (6.1%)	0.605
Non-fatal MI	3 (6.3%)	4 (8.2%)	5 (10.2%)	0.778
Arrhythmias	3 (6.3%)	3 (6.1%)	6 (12.2%)	0.453
Heart failure	4 (8.3%)	2 (4.1%)	7 (14.3%)	0.205
Non-fatal stroke	1 (2.1%)	1 (2%)	2 (4.1%)	0.779
LVEF at 6 months (%)	55 (50–59)	53 (44–58)	49.5 (40–55)	0.009
Change in LVEF (%)	11 (7–16)	8 (5–15)	5 (1.5–10)	0.001

## Discussion

To date, few studies have explored the potential benefit of withholding stent implantation in acute myocardial infarction when primary percutaneous coronary intervention (PCI) strategy was chosen [29].

Accordingly, this study was conducted aiming to compare the effects of immediate versus deferral of stenting and explore the benefits of delaying stenting in 1ry PCI and the better duration of delay after recanalization of the infarct related artery in a subset of patients with heavy thrombus burden.

We have found that the incidence of no/slow-reflow was significantly higher in the immediate stenting group as the percentage of patients with final TIMI flow grade 3 was significantly higher in both deferred stenting groups (group A and B) than in immediate stenting (group C) (82.2% vs. 76%, respectively), the same was found with the percentage of patients with final MBG grade 3. Also, in the DEFER-STEMI study, deferral of stenting 4–16 h caused a reduction in occurrence of no-reflow when compared to immediate stenting (5.9% vs. 28.6%) [16]. This was also consistent with Tang et al. [30] Who found a significant reduction in thrombus burden prior to stenting after 7 days of deferral, with subsequent higher percentage of final TIMI III flow in the deferral group after stenting.

In their landmark trial, DANAMI-3-DEFER, Kelbæk et al. [19] in 2016 tested deferral of stenting in a study population of patients undergoing 1ry PCI randomized to either immediate or deferred stenting, and it showed no benefit and resulted in a class III recommendation for routine deferral of stenting in the latest guidelines.

In the current study, and others’ as DEFER-STEMI trial [16], the population study selected had heavy thrombus burden (TIMI thrombus grade ≥ 2), which we thought would be the subset of patients that would benefit from stent implantation deferral. 56% in our patients’ population had TIMI grade 4 thrombus and 31% had TIMI grade 5 thrombus burden, with no significant difference between the 3 studied groups regarding neither baseline thrombus grade nor TIMI flow in the IRA. The angiographic findings of our trial showed that after the 1st procedure; the percentage of patients with TIMI flow grade 3 was significantly higher in groups A and B compared to group C (90% and 92% vs. 76%, *p* value = 0.034) Also thrombus regression was evident in patients’ 2nd coronary angiography, with nearly half of the patients presented at 2nd procedure with thrombus grade 2. We have also found that regarding the thrombus resolution at the 2nd procedure; group B (7 days deferral) was significantly better than group A (4–16 h deferral), this delineates the only merit of the prolonged interval of deferral in group B, leaving a longer time for the drugs to act on and help in thrombus resolution. However, after the 2nd procedure no significant difference was found between the two groups regarding the TIMI flow as well as the MBG.

In the current study, median lesion length was significantly higher in group C compared to each of groups A and group B. This was consistent with other studies as DEFER-STEMI trial [16] which showed reduced lesion length after stent deferral, however no significant difference was found between groups A and B. This is mostly caused by the spontaneous (auto-thrombolysis) and the pharmacologic induced angiographic changes in the vessel with time after deferral of stenting, resulting in shorter implanted stents with better long-term prognosis and lower rates of in-stent restenosis.

In the current study, in group A (4–16 h deferral), stenting of the IRA was avoided in 4 (8%) patients, while in group B (7 days deferral), stenting was avoided in 10 (20%) patients, which can be explained by the thrombus resolution that occurred after deferral with the subsequent relief of the vasospasm that occurs with the heavy thrombus burden.

In DEFER-STEMI trial [16] stenting was deemed unnecessary in 3 patients in the deferral group representing 6%, also in DANAMI-3-DEFER [19], despite the negative overall results of the Routine deferral of stenting, it showed that in the deferral group stenting was deemed unnecessary in 15% of patients.

While for the other secondary endpoints, the current study showed that regarding the in-hospital outcome; no significant difference was found between the 3 groups regarding over-all MACE, bleeding and CIN. While in respect to 6-months follow-up group C (immediate stenting) showed significantly higher risk of developing MACE (composite endpoint) than group A (4–16 h deferral) (34.7% vs. 14.6%; adjusted HR, 2.59; 95% CI, 1.07–6.25; *p* = 0.034), also the same group showed significantly higher risk of developing MACE than group B (7 days deferral) (34.7% vs. 16.3%; adjusted HR, 2.43; 95% CI, 1.05–5.64; *p* = 0.038). There was no statistical difference in the risk of developing MACE between groups A and B during the follow-up period. The most frequent event reported in group C was heart failure, which can be explained by the lesser percentage of achievement of TIMI grade 3 flow after revascularization of the IRA in comparison with the deferral groups.

In Tang et al. [30], no MACE occurred during period of hospitalization in both groups. After 6 months follow up, there was no significant difference in the occurrence of MACE, but lower incidence of heart failure in the deferral group was noticed (5.0% vs. 19.1%), which is consistent with our study as mentioned.

This was also associated in the current study with the fact that the median LVEF at 6 months was significantly lower in group C (immediate stenting) compared to each of groups A and B (deferred stenting) *p* = 0.021, however no significant difference was found between groups A and B. And the median change in LVEF between the initial echocardiography and the one performed 6 months later was significantly lower in group C compared to groups A and B, however no significant difference was found between groups A and B. In DANAMI-3-DEFER trial [19], an improvement in LVEF at 18 months with deferred stenting was observed in a smaller subset that underwent imaging. While in DEFER-STEMI trial [16], regarding the MRI findings; when compared with immediate stenting, myocardial salvage (percentage of left ventricular mass) (19.7% [IQR: 13.8% to 26.0%] vs. 14.7% [IQR: 8.1% to 23.2%]) and salvage index (68% (IQR: 54% to 82%) vs. 56% (IQR: 31% to 72%)) were higher in the deferred stenting arm after 6 months.

Improved angiographic outcomes provided by delayed stenting, such as the higher percentage of post-PCI TIMI grade 3 flow, have been associated with a reduction in death, myocardial infarction and repeat revascularization after PCI [31]. A potential mechanical explanation for the improvement associated with delayed stenting may actually be the reduction in thrombus burden. In all the studies where quantitative coronary analysis was performed a significant reduction in thrombus burden was observed before and after the interval required for delayed stenting [30].

In the light of the results of our study, and other former studies that we mentioned, deferral of stenting is proved to be a valuable strategy in primary PCI patients with heavy thrombus burden, selection of the candidates is of great importance as DANAMI-3-DEFER [19] proved that routine deferral of stenting in primary PCI was of no value. But our study proves among others that it could be used as a bailout strategy in patients with heavy thrombus burden with resulting improved coronary flow, myocardial recovery, and reduction in lesion length or even the mere need for stent implantation.

## Study limitations

The current study had the following limitations:

1. The sample size was rather small.
2. The study was conducted by several operators.
3. The patients’ and angiographic characteristics might have influenced the operator to perform delayed stenting.
4. Also, cardiac MRI which was not available is a better tool for quantification of left ventricular function changes and the effects of microvascular obstruction.

## Conclusions

This study suggests that; in a selected population of patients undergoing 1ry PCI, with angiographically evident high thrombus burden, stent deferral was proved to be better than immediate stenting after recanalization of IRA, in achieving TIMI III flow, reducing risk of 6 months MACE, and restoration of myocardial function. Longer deferral interval when compared to a strategy of deferral within 24 h, did not prove to be of additional value, except for higher percentage of thrombus resolution before second procedure, which was not reflected on neither final coronary flow nor following clinical events and myocardial recovery with an extra added financial burden. So, stent deferral can be used as a bailout strategy in patients with heavy thrombus burden with resulting improved coronary flow, myocardial recovery, and reduction in lesion length or even the mere need for stent implantation.

## Data Availability

All data generated or analysed during this study are included in this published article [and its supplementary information files].

## Abbreviations

CABG: Coronary artery bypass graft
CIN: Contrast-induced nephropathy
DBP: Diastolic blood pressure
DM: Diabetes mellitus
ECG: Electrocardiogram
EF: Ejection fraction
GIT: Gastro-intestinal tract
HTN: Hypertension
IHD: Ischemic heart disease
IRA: Infarction related artery
LVEF: Left ventricular ejection fraction
MACE: Major adverse cardiovascular events
MBG: Myocardial blush grade
MI: Myocardial infarction
PCI: Percutaneous coronary intervention
RCTs: Randomized control trials
SBP: Systolic blood pressure
SD: Standard deviation
STEMI: ST-segment elevation myocardial infarction
TIMI: Thrombolysis in myocardial infarction
